# Clinical Relevance of Transjugular Liver Biopsy in Comparison with Percutaneous and Laparoscopic Liver Biopsy

**DOI:** 10.1155/2009/947014

**Published:** 2009-11-15

**Authors:** Max G. Beckmann, Matthias J. Bahr, Johannes Hadem, Martin Bredt, Heiner Wedemeyer, Andrea S. Schneider, Stefan Kubicka, Michael P. Manns, Christian P. Strassburg, Jochen Wedemeyer

**Affiliations:** ^1^Department of Gastroenterology, Hepatology and Endocrinology, Medical School Hannover, Carl Neuberg Straße 1, 30625 Hannover, Germany; ^2^Department of Gastroenterology, Sana clinic Lübeck, Kronsorder Allee 17-73, 23560 Lübeck, Germany; ^3^Department of Pathology, Medical School Hannover, Carl Neuberg Straße 1, 30625 Hannover, Germany; ^4^Integrated Research and Treatment Center-Transplantation (IFB-Tx), Medical School Hannover, Carl Neuberg Stra. 1, 30625 Hannover, Germany

## Abstract

*Background*. Transjugular liver biopsy (TJLB) is frequently used to obtain liver specimens in high-risk patients. However, TJLB sample size possibly limits their clinical relevance. *Methods*. 102 patients that underwent TJLB were included. Clinical parameters and outcome of TJLB were analyzed. Control samples consisted of 112 minilaparoscopic liver biopsies (mLLBs) and 100 percutaneous liver biopsies (PLBs). *Results*. Fewer portal tracts were detected in TJLB (4.3 ± 0.3) in comparison with PLB (11.7 ± 0.5) and mLLB (11.0 ± 0.6). No difference regarding the specification of indeterminate liver disease and staging/grading of chronic hepatitis was observed. In acute liver failure (*n* = 32), a proportion of hepatocellular necrosis beyond 25% was associated with a higher rate of death or liver transplantation. *Conclusions*. Despite smaller biopsy samples the impact on the clinical decision process was found to be comparable to PLB and mLLB. TJLB represents a helpful tool to determine hepatocellular necrosis rates in patients with acute liver failure.

## 1. Introduction

Liver biopsy is regarded as the “gold standard” for the evaluation of liver disorders and the most specific test to assess the nature as well as the grading and staging of certain liver diseases [1, 2].

Various clinical conditions require a diagnostic liver biopsy. These include liver disease of unknown origin, acute or subacute liver failure, cellular rejection in patients after liver transplantation, or grading and staging of chronic hepatitis.

Percutaneous liver biopsy (PLB), transjugular liver biopsy (TJLB), and minilaparoscopic liver biopsy (mLLB) are most frequently applied techniques to obtain liver specimens [3, 4].

PLB is usually used as the preferred method, because it is an easy to perform, cost efficient, and reliable method that produces biopsy cylinders of up to 4 cm. However ascites and/or significant coagulopathy preclude the utilization of PLB due to the risk of severe hemorrhage [2, 4]. TJLB is an alternative to PLB, because bleeding resulting from the biopsy needle will drain into the hepatic veins. Other indications for TJLB include massively adipose patients [5]. Transjugular liver biopsy (TJLB) was first experimentally applied in dogs in 1964 [6] and was introduced into clinics in the early 1970s [7]. However, TJLB has been considered to be less satisfactory in comparison with PLB because the samples obtained are smaller and the cylinders thinner owing to the limited size of the introducer set. The introduction of true cut systems has improved the biopsy quality of TJLB in comparison with aspiration biopsies [8–10].

Recently, mLLB has been more widely employed as an alternative to TJLB in cases of coagulopathy and ascites [4, 11]. In contrast to PLB and TJLB, mLLB also provides macroscopic information on the liver surface in addition to histopathology. This way mLLB can help to avoid sampling errors. In addition bleeding from biopsy puncture sites can be coagulated under direct vision.

The goal of this study was to elucidate the clinical relevance of the TJLB with respect to specific clinical indications (indeterminate liver disease, acute/subacute liver failure, chronic hepatitis, and suspected graft rejection after liver transplantation). Furthermore, the significance of the TJLB for the specific clinical problem was compared with results from PLB and mLLB.

## 2. Materials and Methods

We identified all patients that underwent TJLB at the Hannover Medical School between January 2000 and October 2007. As controls we selected a cohort of 100 consecutive patients that underwent PLB from 01/2007–06/2007. As a third group, all patients that underwent mLLB in the period 04/2005–10/2007 served as controls.

Complete patient charts were available in electronic format. The following data were extracted. There is indication for either PLB, TJLB, or mLLB. Indications were grouped as:

1. diagnostic work up for liver disease of unknown origin,
2. Staging and grading of chronic hepatitis,
3. diagnostic work up for acute/subacute liver failure,
4. diagnostic work up of liver transplant (oLT) patients with elevated liver function tests (LFTs),
5. others, for example, cholestatic liver disease.

Contraindications for PLB were recorded: (i) presence of ascites, (ii) coagulopathy (defined as prothrombin time >50% above normal and/or partial thromboplastin time >50 seconds and/or platelet count <50 10^3^/*μ*L), or (iii) presence of ascites and coagulopathy. The technical feasibility as well as the reasons for unsuccessful biopsy were extracted from the biopsy protocols. Complications related to the biopsies and clinical consequences were detected from the biopsy protocols and by reviewing the patients' charts.

To assess the quality of the biopsy specimen several parameters were recorded including the number of needle passes, insufficient biopsy material according to the pathological report, and the number of complete portal tracts. If the number of complete portal tracts was not specified in the report, stored biopsy slides were re-evaluated (M.G.Beckmann and M.Bredt).

To assess the impact of the biopsy on the clinical course the pathology report and the discharge summary as well as the long-term clinical course were reviewed (J.Wedemeyer). For the group of patients that underwent biopsies to evaluate idiopathic/cryptogenic liver disease we determined from the patients' charts whether a specific diagnosis could be established or whether liver disease remained cryptogenic. If a specific diagnosis was established, we evaluated whether the histological results from the biopsies either were relevant for the establishment of the diagnosis or whether no specification was given in the histological report that supported the final diagnosis.

In the patients that underwent TJLB for staging/grading of chronic hepatitis we checked the pathology reports whether a histological staging/grading according to Ishak et al. [12] was possible.

In the group of patients that were evaluated for acute/subacute liver failure we additionally reviewed the proportion of necrosis of the liver parenchyma. Necrosis was grouped as follows: (1) no necrosis, (2) low necrosis (1%–25%), (3) medium necrosis (26%–50%), (4) severe necrosis (51%–75%), and (5) complete necrosis (76%–100%). If necrosis was not quantified in the pathology report, stored slides were reevaluated (M.Bredt). Additionally, we assessed the clinical outcome in this patient group. Outcome was categorized as (1) spontaneous recovery, (2) liver transplantation, and (3) death.

### 2.1. Biopsy Procedures

All types of biopsy procedures were carried out according to standardized protocols.

Most of the TJLB (*n* = 99, 97%) and all mLLB were performed under conscious sedation using midazolam and disoprivan. No sedation related complications were reported. The sedation was performed and controlled by the physicians with support of the nursing staff. In 3 cases TJLB was performed in general anesthesia, because the patient was already intubated due to progressive encephalopathy and/or respiratory failure before the decision to perform transjugular liver biopsy was made.

Blood pressure, heart rate, and oxygen saturation were monitored noninvasively. For TJLB continuous ECG monitoring was used.

TJLB was performed via the right jugular vein. After local anaesthesia and puncture of the right jugular vein a 0.89 mm diameter Terumo guide wire (Terumo, Eschborn, Germany) was inserted. In all TJLB procedures, an 18 G Quick—Core biopsy set (true cut system, Cook Medical, Denmark) was used, which was introduced via the guide wire. Correct positioning of the biopsy set was verified by fluoroscopy. In 2006, ultrasonography of the jugular vein before puncture was implemented in the routine protocol.

For percutaneous biopsy the position and direction of the biopsy tract were defined under ultrasound control. Five mL xylocaine (1%) was used as local anesthesia. All biopsies were performed using a Menghini 17 G aspiration needle (Gallini, Mantova, Italy). Safety controls included the determination of haemoglobin 4 hours after biopsy.

For laparoscopy a pneumoperitoneum was created by N_2_O insufflation via a Veress needle in general to the left and lateral of the umbilicus. A second access was obtained on the right side by inserting a second trocar. A 16 G true cut needle (BIP GmbH, Tuerkenfeld, Germany) was inserted and biopsies of the liver were taken from the left and right lobes under vision. Biopsy sites were prophylactically coagulated.

### 2.2. Statistics

Data obtained from the patient charts were analyzed using the software Statview Version 5.0.1. The various groups were compared using *χ* ^2^-test, correlation *z*-test or Student's *t*-test as appropriate. A *P*-value below .05 was considered significant.

## 3. Results

### 3.1. Transjugular Liver Biopsy Patients

101 patients underwent 102 TJLB. Forty five (45%) patients were female; the median age was 47 years (range 16–75). Contraindications for PLB that led to TJLB were coagulopathy (*n* = 42/41%), ascites (*n* = 33/32%), or combined ascites and coagulopathy (*n* = 27/27%). Indications for liver biopsy were a diagnostic work up for indeterminate liver disease (*n* = 45/44%), acute/subacute liver failure (*n* = 32/31%), staging/grading of chronic hepatitis (all: *n* = 12/12%, hepatitis C [HCV]: *n* = 6/5.9%, hepatitis B [HBV]: *n* = 3/2.9%, autoimmune hepatitis [AIH]: *n* = 3/2.9%), diagnostic work up of OLT patients with elevated LFTs (*n* = 11/10.8%), and others (*n* = 2/2%, PBC/malignant tumour) (Table 1).

### 3.2. Percutaneous Liver Biopsy Patients

One hundred consecutive PLBs in 100 patients were evaluated. The median age was 47 years (range 18–77). Females and males were equally distributed (50 female, 50 male). Indications for performing PLB were a diagnostic work up for indeterminate liver disease (*n* = 32/32%), staging/grading of chronic hepatitis (all: *n* = 43/43%, HCV: *n* = 26/26%, HBV: *n* = 12/12%, AIH: *n* = 5/5%), a diagnostic work up of OLT patients with elevated LFTs (*n* = 19/19%), acute/subacute liver failure (*n* = 1/1%), and others (all: *n* = 5/5%, primary sclerosing cholangitis [PSC]: *n* = 2/2%, primary biliary cirrhosis [PBC]: *n* = 2/2%, alpha-1 antitrypsin deficiency: *n* = 1/1%) (Table 1).

### 3.3. Laparoscopic Liver Biopsy Patients

mLLB was performed in 112 patients. The median age was 46 years (range 17–75). 49 (44%) of the patients were women. Fifteen patients fulfilled contraindication criteria for PLB (*n* = 13/12% ascites, *n* = 2/2% ascites and coagulopathy). Indications for performing mLLB were a diagnostic work up for indeterminate liver disease (*n* = 38/34%), staging/grading of chronic hepatitis (all: *n* = 24/21%, HCV: *n* = 17/15%, AIH: *n* = 5/5%, HBV: *n* = 2/2%), others (all: *n* = 50/45%, PSC: *n* = 33/30%, PBC: *n* = 5/5%, malignant tumour: *n* = 10/9%, alpha-1 antitrypsin deficiency: *n* = 2/2%) (Table 1).

### 3.4. Technical Success of TJLB

84 (82%) of 102 TJLB were successful. In 10 (10%) cases investigators failed to cannulate a hepatic vein, in 6 (6%) cases the investigators could not pass the right atrium, cannulate the jugular vein, or access the superior vena cava. In 2 (2%) cases cannulation of the hepatic vein was successful, but despite repeated attempts no sufficient biopsy material could be obtained.

Cannulation of the hepatic vein failed in 2 of the 11 (18%) patients that presented with elevated LFTs after OLT. In contrast, in the other indication groups the cannulation of the hepatic veins failed in 9% (*n* = 4) of patients that presented diagnostic work up for indeterminate liver disease, 9% (*n* = 3) of patients with acute/subacute liver failure and 8% (*n* = 1) of patients with chronic hepatitis. This difference was not statistically significant (*χ* ^2^-test; *P* = .9).

All PLBs were technically successful and 111 of 112 (99%) mLLB were technically successful. In one case mLLB failed due to an obstructed visualization of the liver surface.

The technical success rate was significantly lower in TJLB (82%) than in PLB or mLLB (*χ* ^2^-test; *P* = .0005).

### 3.5. Complications

In the TJLB group no major complications according to SIR (Society of Interventional Radiology) guidelines were observed [13]. In one case, an intrainterventional rupture of the liver capsule was suspected, which was not confirmed during the subsequent clinical course. In another case, the carotid artery had been accidentally punctured. However, no further bleeding or infectious complications occurred. In one case, injection of contrast agent to confirm the correct position of the introducer set resulted in formation of a radiopaque depot without further consequences. In total, this corresponds to a minor complication rate of 2.9%.

The complication rate of the PLB was 3% In one case, the patient developed a vasovagal syncope treated with fluid substitution. Furthermore, we observed one case of severe postinterventional pain that required treatment with opioid analgetics and one case with a drop in hemoglobin in the 4 hours postinterventional control. No substitution of blood products was required.

After mLLB complications were observed in 3 patients (2.7%). One was a major complication with postinterventional bleeding that required substitution with blood products. The other two complications were minor including a biliary leak and minor bleeding from the biopsy location. Both could be handled with coagulation during the intervention. No substitution with blood products was required in this case.

### 3.6. Quality of Biopsy Specimens

With TJLB a median of 2 biopsies (range 1–7), with PLB a median of 1 biopsy (range 1-2), and with mLLB a median of 2 biopsies (range 1–7) were taken.

**Figure 1:**
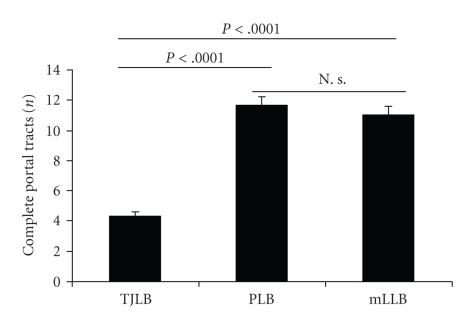
*Biopsy quality*. Significantly more portal tracts were detected in PLB and MLLB if compared to TJLB.

On average, 4.3 ± 0.3 complete portal tracts (range 0–12) were identified in TJLB samples (Figure 1). In 14 (16%) cases, biopsies were judged as inappropriate for complete evaluation in the pathology report (indeterminate liver disease [*n* = 10], LFT elevation after OLT [*n* = 2], chronic hepatitis [*n* = 1], others [*n* = 1]). These specimens had an average of only 1.6 ± 0.4 (range 0–5) detectable complete portal tracts.

A calculated mean of 11.7 ± 0.5 and of 11.0 ± 0.6 complete portal tracts was found in PLB and mLLB, respectively (Figure 1). In 4 cases of both PLB and mLLB biopsy specimens were inappropriate for complete evaluation. These biopsies contained a mean of 3.9 ± 0.8 and 7.5 ± 1.3 portal tracts.

With regard to number of portal tracts (*P* < .0001, Student's *t*-test; see Figure 1) and the appropriateness for histological evaluation (*P* < .05, *χ* ^2^-test), the quality of the TJLB samples was significantly lower compared to that PLB and mLLB.

### 3.7. Indeterminate Liver Disease

One of the main indications for a liver biopsy in liver disease of unknown etiology is to establish a diagnosis allowing an appropriate therapy. Furthermore, biopsy may reveal prognostic information (e.g., fibrosis versus cirrhosis).

**Figure 2:**
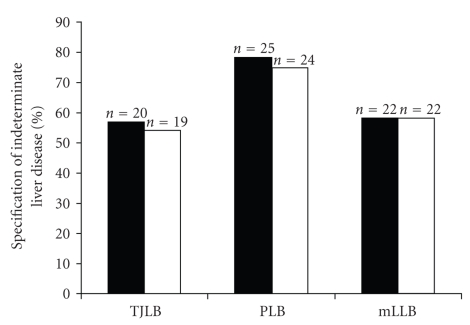
*Indeterminate liver disease*. Black bars show the percentage of cases in which the etiology of liver disease was identified during the clinical course. The white bars show the percentage of cases in which liver biopsy contributed substantially to the establishment of the diagnosis. No difference was found between the rate of TJLB, PLB and mLLB.

In the TJLB group, specification of the underlying liver disease was possible in 57% (*n* = 20/35) of patients during the clinical course. In 54% the TJLB was relevant to the establishment of the diagnosis (Figure 2).

In the PLB group a specification of disease was reached in 78% (*n* = 25) and PLB contributed to the diagnosis in 75% (*n* = 24) of patients with etiologically unclear liver disease (*n* = 32), while in the mLLB group (*n* = 38) a specification was successful in 58% (*n* = 22) and histology contributed in all cases (*n* = 22) (Figure 2).

Statistical analysis revealed no significant difference between the three groups in respect to specification and the impact of the histology (*χ* ^2^-test, Figure 2).

### 3.8. Acute/Subacute Liver Failure

**Figure 3:**
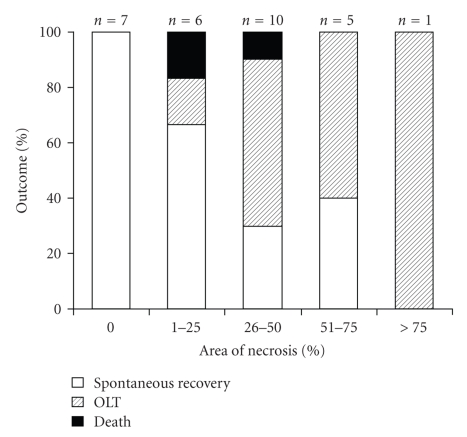
*Hepatocellular necrosis in acute/subacute liver failure.* More than 25% of hepatocellular necrosis was associated with a poor transplant free survival.

The underlying disease could be clarified in 51% (*n* = 15) of patients who underwent TJLB for acute/subacute liver failure. No necrosis was found in 7 cases, low grade (1%–25%) necrosis was detected in 6, medium grade (26%–50%) necrosis was detected in 10, severe (51%–75%) in 5, and complete necrosis (76%–100%) was found in 1 case. Spontaneous recovery was observed in 83% of patients with 0%–25% necrosis, while only 31% of patients that had more than 25% of necrosis survived without liver transplantation. In general, higher grades of necrosis were associated with a significantly lower proportion of transplant free survival (*χ* ^2^-test, *P* < .036) (Figure 3).

In 22 (69%) of the cases with acute/subacute liver failure the underlying disease could be clarified during the clinical course. We found steatohepatitis in 5, malignant tumour in *n* = 3, viral hepatitis *n* = 3, autoimmune hepatitis in *n* = 4, Wilson's disease in *n* = 1, hemochromatosis in *n* = 1, and toxic reaction in *n* = 5 cases. In *n* = 15 (52%) the TJLB was relevant to the establishment of the diagnosis. A specific therapy was started in *n* = 6 (19%) of patients. Interestingly, TJLB contributed to the diagnosis in all these cases.

Unfortunately, we could not generate a sufficient control group for acute/subacute liver failure from the PLB (*n* = 1) and mLLB (*n* = 0) groups.

### 3.9. Chronic Hepatitis

**Figure 4:**
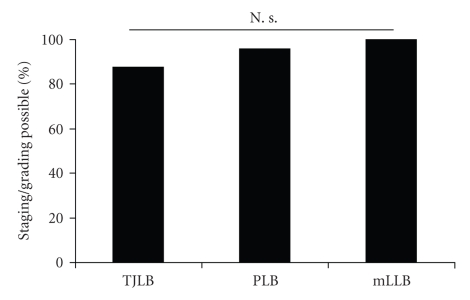
*Staging/grading in chronic hepatitis*. No statistically significant difference in the rate of staging/grading for chronic hepatitis was detected between the three groups.

To guide antiviral and immunosuppressive therapy in chronic hepatitis an adequate grading and staging is essential. The biopsy specimen was appropriate for scoring in 88% (*n* = 7) of patients with chronic hepatitis that underwent TJLB (Figure 4).

In the chronic hepatitis group, sufficient scoring was possible with PLB in 98% (*n* = 42) and with mLLB in 100% (*n* = 24, Figure 4).

We did not find a significant difference between the three groups (*χ* ^2^-test; *P* = .156). However, the low number of patients in the TJLB group (*n* = 8) should be noted.

### 3.10. Diagnostic Work up of Liver Transplant Patients with Elevated LFTs

Histological analysis in this patient group using TJLB revealed cholestasis (*n* = 3), hepatitis (*n* = 1), ischemia associated alteration (*n* = 1), chronic rejection (*n* = 1), or unspecified alterations (*n* = 2). In all cases histological results were important for clinical management as histology ruled out acute rejection in all cases. Due to adhesions no mLLB was performed in this patient group.

### 3.11. Discussion

In our patient cohort, TJLB was associated with a low minor complication rate of 2.9%. No major complications occurred. In comparison with PLB and mLLB, no significant difference in intervention associated risks was observed despite significant coagulopathy in 68% of the patients that underwent TJLB. In this regard, our data are comparable to the complication rates reported by other groups. In their systematic review of 64 publications including 7649 TJLBs, Kalambokis et al. observed minor complications in 6.5% and major complications in 0.56% of patients [5]. Therefore, despite the significantly impaired liver function, in most cases TJLB can clearly be considered a low-risk intervention.

However, our data analysis also showed a high rate of technical failure in the TJLB group. With only 82% technical success we were less effective than other groups with reported technical success rates between 87% and 100% also using a true cut biopsy set as in our clinic. The reason for this high rate of technical failure especially the high rate of failed cannulation of the hepatic vein is not clear. We suspected that the high rate of transplant patients and their altered vascular anatomy may have influenced the outcome in our study [14]. Indeed, the cannulation of a hepatic vein failed more often in patients that underwent liver transplantation than in the other patient groups. However, this difference was not statistically significant. Furthermore, other groups recently reported no technical problems with TJLB in transplant patients [15, 16]. Another reason may be the low annual rate of 15 TJLBs/year performed at our hospital in comparison with other groups that reported annual rates between 48 and 111 [17]. In parallel to our evaluation Soyer et al. recently suggested the use of ultrasonographic guidance [18]. Since 2006, we consequently applied ultrasonographic guidance for jugular vein puncture for TJLB. Fortunately, no technical failures have occurred from 04/2006 until today at our institution.

A liver biopsy cylinder of ≥15 mm length containing 6–10 complete portal tracts is regarded sufficient for the histological diagnosis of diffuse liver diseases [1, 19, 20], whereas 20 mm and/or 11 portal fields are regarded as minimum for a meaningful grading and staging in chronic hepatitis [21, 22]. It was therefore not surprising that the quality of TJLB turned out to be significantly lower than biopsies generated from PLB or mLLB if judged by the pathologist's evaluation and the number of portal tracts. We found a mean of 4.3 ± 0.3 complete portal tracts in our TJLB samples. In their analysis, Kalambokis et al*.* reported a mean of 6.8 complete portal tracts [5]. However, despite the significantly lower number of complete portal tracts in comparison with PLB and mLLB, we were not able to find a difference in terms of staging/grading for chronic hepatitis. Furthermore, in the indeterminate liver disease group the proportion of cases in which a liver biopsy contributed to the establishment of a diagnosis was not significantly different between TJLB, PLB and mLLB. However, it has to be emphasized that the contribution of a biopsy sample to the establishment of a diagnosis is difficult to evaluate and therefore the statistical analysis has to be judged carefully. Furthermore, understaging and sampling error resulting from small biopsy cylinders cannot be excluded [21]. In addition the low success rate of TJLB in our cohort is another disadvantage and might affect the ability of diagnosis using TJLB. Therefore, we feel that especially in patients with ascites mLLB is a valuable alternative to TJLB. Our study shows that biopsies generated from mLLB are of significantly better quality. In addition, visualization of the liver surface in mLLB gives additional information and avoids sampling errors [4, 23]. So far, only few data have been reported evaluating mLLB as an alternative to TJLB in patients with severe coagulopathy [11]. If mLLB is not available a minimum of three needle passes with TJLB should be performed to obtain optimal tissue samples [24]. Cholongitas et al*.* did not find a difference in the number of portal tracts if at least three needle passes were applied in comparison with PLB using a Menghini needle [15, 24]. This group even suggested four needle passes in their most recent publication [25].

Acute/subacute liver failure is a dramatic and potentially fatal condition characterized by severely impaired liver function with jaundice, coagulopathy and encephalopathy. The underlying etiologies include toxic, infectious, autoimmune, and metabolic etiologies. Therefore, a timely diagnosis needs to be established to begin specific therapy whenever indicated or to evaluate emergency liver transplantation. Variables that have been used to predict prognosis are hepatic encephalopathy, serum bilirubin, and coagulopathy. Furthermore scores such as the King's College criteria have been established to estimate the prognosis in patients with acute liver failure [26]. Liver histology may be supportive in this clinical situation because it can estimate the grade of necrosis and hopefully the regenerative activity. However, the severe coagulopathy associated with acute liver failure hinders regular percutaneous liver biopsies in this clinical setting. Therefore, TJLB is an important diagnostic tool in acute liver failure with severe coagulopathy. Despite a vast amount of studies employing TJLB, only two studies have been published so far that have focussed on the role of TJLB in acute liver failure [27, 28]. Donaldson and colleagues retrospectively investigated 60 successful transvenous liver specimens from patients with acute liver failure. They found a correlation between degree of hepatocellular necrosis and survival. Especially a necrosis rate of greater than 70% was associated with a poor outcome. Miragli et al*. *prospectively studied 17 patients who underwent TJLB for acute liver failure. They showed that TJLB can alter an initial diagnosis (18% of cases). Furthermore, necrosis of less than 60% was associated with a better prognosis than submassive or massive necrosis (≥85%), which resulted in death or liver transplantation. Our study is the second largest cohort (*n* = 32) that studied the relevance of TJLB in acute liver failure. In accordance with the studies by Donaldson et al*.* and Miragli et al*.* we were able to show that higher rates of hepatocellular necrosis are related to higher rates of death and/or liver transplantation. A proportion of necrosis of more than 25% was already associated with a significantly lower rate of organ/patient survival. However, as the degree of necrosis might have influenced the decision to perform liver transplantation, the predictive value of hepatic necrosis for patient outcome in acute liver failure has to be interpreted cautiously. The fact that we found a slightly better outcome in the 51%–75% group than in the 26%–50% group (although statistically not significant) may be attributed to a non avoidable sampling error.

## 4. Conclusion

We conclude from our retrospective study that liver biopsies generated via the transjugular approach are indeed valuable for clinical decisions in various indication groups, although sample quality may limit the diagnostic power in some patients. Furthermore, the degree of hepatic parenchymal necrosis which can be estimated by means of TJLB might predict outcome in patients with acute/subacute liver failure. We therefore suggest performing TJLB in patients with acute liver failure that are possible liver transplant candidates to estimate prognosis. In accordance with recent studies, a minimum of four needle passes should be performed to optimize sample quality.

## Figures and Tables

**Table 1:** Distribution of different patient groups to liver biopsy methods applied.

Indication	TJLB	PLB	LLB
Indeterminate liver disease (*n*)	45 (44%)	32 (32%)	38 (34%)
Acute/subacute liver failure (*n*)	32 (31%)	1 (1%)	0 (0%)
Chronic hepatitis	12 (12%)	43 (43%)	24 (21%)
LFT elevation after OLT (*n*)	11 (11%)	19 (19%)	0 (0%)
Others (*n*)	2 (2%)	5 (5%)	50 (45%)
Total (*n*)	102	100	112
